# Hypertension Subtypes, Mortality Risk, and Differential Effects Between Two Hypertension Guidelines

**DOI:** 10.3389/fmed.2022.814215

**Published:** 2022-07-05

**Authors:** Hui Mai, Chao Li, Kangyu Chen, Zhenqiang Wu, Xuanyi Liang, Yongjuan Wang, Tao Chen, Fengjian Chen

**Affiliations:** ^1^Department of Neurology, Central People's Hospital of Zhanjiang, Zhanjiang, China; ^2^Department of Epidemiology and Health Statistics, School of Public Health, Xi'an Jiaotong University Health Science Centre, Xi'an, China; ^3^Division of Life Sciences and Medicine, Department of Cardiology, The First Affiliated Hospital of USTC, University of Science and Technology of China, Hefei, China; ^4^Department of Geriatric Medicine, The University of Auckland, Auckland, New Zealand; ^5^Department of Public Health, Policy and Systems, Institute of Population Health, Whelan Building, Quadrangle, The University of Liverpool, Liverpool, United Kingdom

**Keywords:** mortality, hypertension guideline, cardiovascular risk, hypertension subtype, prognostic risk, cohort study

## Abstract

**Aim:**

To examine which hypertension subtypes are primarily responsible for the difference in the hypertension prevalence and treatment recommendations, and to assess their mortality risk if 2017 American College of Cardiology (ACC)/American Heart Association (AHA) hypertension guideline were adopted among Chinese adults.

**Methods:**

We used the nationally representative data of China Health and Retirement Longitudinal Study (CHARLS) to estimate the differences in the prevalence of isolated systolic hypertension (ISH), systolic diastolic hypertension (SDH) and isolated diastolic hypertension (IDH) between the 2017 ACC/AHA and the 2018 China Hypertension League (CHL) guidelines. We further assessed their mortality risk using follow-up data from the China Health and Nutrition Survey (CHNS) by the Cox model.

**Results:**

The increase from the 2017 ACC/AHA guideline on hypertension prevalence was mostly from SDH (8.64% by CHL to 25.59% by ACC/AHA), followed by IDH (2.42 to 6.93%). However, the difference was minuscule in the proportion of people recommended for antihypertensive treatment among people with IDH (2.42 to 3.34%) or ISH (12.00 to 12.73%). Among 22,184 participants with a median follow-up of 6.14 years from CHNS, attenuated but significant associations were observed between all-cause mortality and SDH (hazard ratio 1.56; 95% CI: 1.36,1.79) and ISH (1.29; 1.03,1.61) by ACC/AHA but null association for IDH (1.15; 0.98,1.35).

**Conclusion:**

Adoption of the 2017 ACC/AHA may be applicable to improve the unacceptable hypertension control rate among Chinese adults but with cautions for the drug therapy among millions of subjects with IDH.

## Introduction

The threshold to define hypertension was changed from ≥140/90 mm Hg in the Eighth Joint National Committee guideline (1) to ≥130/80 mm Hg in the 2017 American College of Cardiology (ACC)/American Heart Association (AHA) hypertension guidelines (2). However, in contrast to the American guideline, China Hypertension League (CHL) guideline (3) continues to recommend a threshold of ≥140/90 mm Hg for diagnosing hypertension, which is in line with the 2018 European Society of Cardiology (ESC) guideline (4). As such, the definitions for isolated systolic hypertension (ISH), systolic diastolic hypertension (SDH) and isolated diastolic hypertension (IDH) now differ between China, Europe, and America.

Previous studies have primarily assessed the population impact on the prevalence or number of hypertension according to the 2017 ACC/AHA guideline. As expected, these studies suggested that the new hypertension guideline will substantially increase the number of people labeled having hypertension (5–10). However, few studies have further explored which subtypes (i.e., ISH, SDH or IDH) are primarily responsible for this increase and the impact of the 2017 ACC/AHA guidelines on antihypertension therapy treatment by these hypertension subtypes. Additionally, evidence has shown that different hypertension subtypes may have different prognostic implications and require different therapeutic management. Therefore, it is vital to investigate the prognostic implication of the newly defined ISH, SDH and IDH from the 2017 ACC/AHA guideline after including those with systolic blood pressure (SBP) of 130–139 mmHg or diastolic blood pressure (DBP) of 80–89 mmHg as having hypertension (11–13).

In the current study, we aimed: (1) to compare the impact of the 2017 ACC/AHA guideline on the prevalence of ISH, SDH and IDH and candidacy for the initiation of antihypertensive treatment and (2) to assess the mortality risk with ISH, SDH or IDH according to the 2017 ACC/AHA or 2018 CHL guidelines.

## Methods

### Setting and Data Sources

We used nationally representative data from the China Health and Retirement Longitudinal Study (CHARLS) to estimate the population impact on different hypertension subtypes using the 2017 ACC/AHA guideline compared to the 2018 CHL guideline. The study design of CHARLS has been previously reported (14). Briefly, CHARLS is an ongoing nationally representative survey in China that recruited 17,708 subjects aged 45 years and older in 2011–2012. The participants were selected through a multistage probability sampling method, which can be weighted to obtain national estimates (details in the Appendix). The CHARLS data that support the findings of this study are available upon application from https://charls.pku.edu.cn/.

We further explored the mortality risk of ISH, SDH or IDH using data from the China Health and Nutrition Survey (CHNS). The cohort profile and data quality of CHNS have been reported elsewhere (15). In summary, the CHNS started in 1989 and was followed up every 2–4 years. Subjects were selected using a multistage random-cluster sampling process from nine provinces across China for all age populations (details in the Appendix). In our study, we restricted our analysis to a population aged over 18 years old. The CHNS data are available upon application from https://www.cpc.unc.edu/projects/china.

For the present analysis, participants from CHARLS and CHNS were included if they were not receiving antihypertensive treatment. The study was approved by the Human Research Ethics Committee of the Xi'an Jiaotong University Health Science Center (No: 2021-6).

### Blood Pressure (BP) Measurement and Categories

Seated SBP and DBP for each participant were measured three times at 45-s intervals using Omron digital devices (Omron model HEM-7200) in CHARLS (14) and 3 times at 30-s intervals using calibrated mercury sphygmomanometers in CHNS (15). For each participant, the mean of 3 BP measurements was used in the current analysis.

Normotension was defined as SBP <140 mm Hg and DBP <90 mm Hg by CHL, and SBP <130 mm Hg and DBP <80 mm Hg by ACC/AHA. Optimal BP was both defined as SBP <120 mm Hg and DBP <80 mm Hg by CHL and ACC/AHA. ISH was defined as SBP in the hypertensive range (CHL: SBP ≥140 mm Hg; ACC/AHA: SBP ≥130 mm Hg) but DBP not in the hypertensive range (CHL: DBP <90 mm Hg; ACC/AHA: DBP <80 mm Hg). Likewise, IDH was defined as DBP in the hypertensive range (CHL: DBP ≥90 mm Hg; ACC/AHA: DBP ≥80 mm Hg) but SBP not in the hypertensive range (CHL: SBP <140 mm Hg; ACC/AHA: SBP <130 mm Hg); SDH was defined as DBP in the hypertensive range (CHL: DBP ≥90 mm Hg; ACC/AHA: DBP ≥80 mm Hg) and SBP in the hypertensive range (CHL: SBP ≥140 mm Hg; ACC/AHA: SBP ≥130 mm Hg) (2, 3).

### Recommendations for Antihypertensive Medication

The 2017 ACC/AHA guidelines recommended initiating antihypertensive medication in subjects with stage 2 hypertension (≥140/90 mm Hg) or stage 1 hypertension (≥130/80 mm Hg) with a history of cardiovascular disease (CVD), age≥65 years, chronic kidney disease (CKD), diabetes, or an expected 10-year risk of atherosclerotic cardiovascular disease (ASCVD) of 10% or greater (2). In our study, we defined the history of CVD as reported coronary heart disease, stroke and/or heart failure; diabetes as a self-reported history of diabetes or current use of diabetic medications or hemoglobin A_1c_ >6.5%; CKD as estimated glomerular filtration rate (eGFR) rate <60 mL/min per 1.73 m^2^ calculated using the 2009 CKD Epidemiology Collaboration equation (16) and the 10-year predicted ASCVD risk calculated using the Pooled Cohort risk equations (17).

The 2018 CHL guideline recommend initiating antihypertensive medication in adults with confirmed hypertension. In addition, antihypertensive medication is recommended in the following situations: diabetes when SBP≥130 mm Hg or DBP≥80 mm Hg and hypertension with age≥65 years when SBP≥150 mm Hg or DBP≥90 mm Hg (3). Detailed comparisons regarding recommendations for antihypertensive medication between 2018 CHL and 2017 ACC/AHA can be found in Appendix Table 1.

### Assessment of Covariables

Body mass index (BMI) was calculated as weight (in kilograms) divided by height (in meters) squared. Categorical covariates included gender (male or female), educational level (illiterate, primary, middle/high school, bachelor degree or above), marital status (married, never), residence status (rural, urban), self-reported health in excellent/very good condition (yes, no), smoking status (non-smoker, current smoker, ex-smoker), drinking status (non-drinker, drinker), and self-reported CVD, diabetes and cancer (yes, no).

### Data Analysis

Using CHARLS sampling weights extrapolated to the Chinese population aged 45 years and older, we first estimated the percentage and absolute number of SDH, ISH or IDH and eligibility of pharmacologic treatment according to CHL and ACC/AHA guideline separately and then compared the differences between guidelines. We then repeated the above analysis by sex and age group. Furthermore, we calculated the percentage of Chinese adults who had concordant and discordant definitions for ISH, SDH or IDH according to the two different sets of guidelines.

We characterized the associations of different hypertension subtypes with total mortality and premature death (defined as mortality before age 73.64 years in men and 79.43 years in women, which was the average life expectancy in China in 2015 (18)) using CHNS data. Baseline characteristics of subjects in the CHNS study were described by normotension, ISH, SDH and IDH groups and are expressed as the mean ± standard deviation and n (%) for continuous and categorical variables, respectively. The cumulative mortality rates according to hypertension subtypes were estimated using Kaplan–Meier (KM) curves and compared using the log-rank test. The Cox proportional hazards model was employed to calculate hazard ratios (HRs) and 95% confidence intervals (CIs) for death associated with each hypertension subtype with normotension as the reference. HRs were adjusted for age, sex, educational level, marital status, rural residents, drinking/smoking status, BMI, self-reported health status, and a history of diabetes mellitus, CVD or cancer, but sensitivity analyses were performed by including the variables sequentially. In addition, we repeated the analysis using the optimal BP group (i.e., SBP <120 mm Hg and DBP <80 mm Hg), which is uniform for both CHL and ACC/AHA guidelines, as the reference in the Cox proportional hazards model. All reported *P*-values are two-sided, and data analyses were conducted using Stata 15.0 software.

## Results

### Percentage and Number of Subjects With ISH, SDH, or IDH

**Figure 1:**
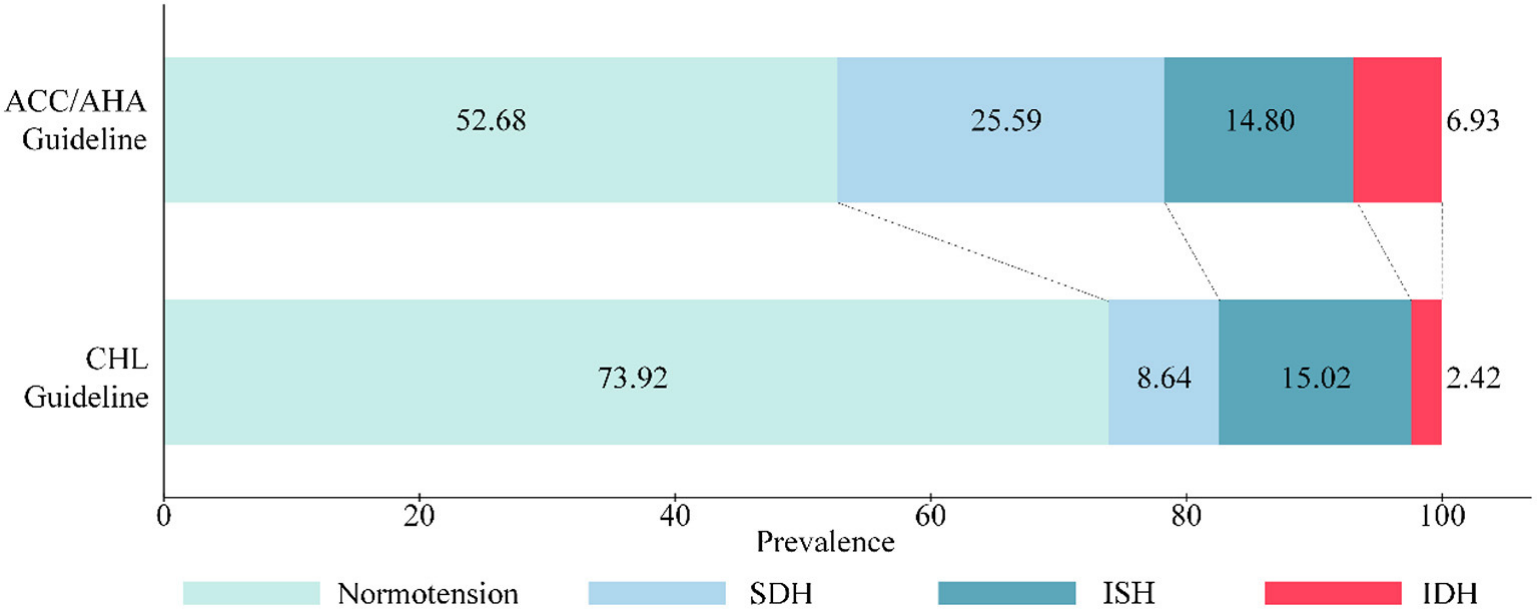
Prevalence of SDH, ISH and IDH defined by ACC/AHA and CHL guidelines among subjects aged ≥45 years.

After excluding 2,578 (18.81%) subjects who were currently receiving antihypertensive treatment from a total population of 17,708 in CHARLS, we determined that the estimated prevalence of hypertension increased from 26.08% when defined by CHL to 47.32% when classified by ACC/AHA. Specifically, this dramatic increase was mostly from the increased prevalence of SDH, from 8.64% (95% CI: 7.51, 9.93) by CHL to 25.59% (23.49, 27.81) by ACC/AHA, followed by the prevalence of IDH, from 2.42% (1.54, 3.77) by CHL to 6.93% (6.02, 7.95) by ACC/AHA. In contrast, the prevalence of ISH was reduced from 15.02% (13.28, 16.94) by CHL to 14.80% (13.33, 16.40) by ACC/AHA, which represented a notably minuscule decrease of 0.22% (−2.37, 2.81) (Figure 1 and Table 1). This pattern was consistently observed across different ages and sexes, although the difference was most pronounced in subjects aged <65 years (5.39 vs. 1.65%) for IDH prevalence and a smaller difference for ISH prevalence (0.05 vs. 0.78%) (Appendix Table 2). The corresponding number of the population of each hypertension subtype and their difference between guidelines was estimated (Table 1 and Appendix Table 2).

**Table 1:** Comparison of Proportion, Numbers of ISH, SDH and IDH, and recommended medication among population aged ≥45 years (from CHARLS 2011 baseline data).

**Description**	**2018 CHL guideline**		**2017 ACC/AHA guideline**		**Difference between CHL and ACC/AHA**	
	**Proportion**	**Numbers (95%CI)**	**Proportion**	**Numbers (95%CI)**	**Proportion**	**Numbers (95%CI)**
	**(95%CI), %**	**million**	**(95%CI), %**	**million**	**(95%CI), %**	**million**
**ISH**						
ISH in total population	15.02	64.18	14.80	63.22	0.22	0.94
	(13.28, 16.94)	(57.29, 71.06)	(13.33, 16.40)	(57.97, 68.47)	(-2.37, 2.81)	(10.12, 12.00)
Recommended antihypertensive	12.00	51.24	11.73	50.10	0.27	1.14
medication in total population	(10.34, 13.88)	(44.38, 58.11)	(10.80, 12.73)	(45.85, 54.34)	(-1.72, 2.26)	(-7.34, 9.65)
**IDH**						
IDH in total population	2.42	10.33	6.93	29.59	−4.51	−19.26
	(1.54, 3.77)	(6.25, 14.41)	(6.02, 7.95)	(26.50, 32.68)	(−5.68, −3.34)	(−24.25, −14.26)
Recommended antihypertensive	2.42	10.33	3.34	14.25	−0.92	−3.92
medication in total population	(1.54, 3.77)	(6.25, 14.41)	(2.64, 4.20)	(11.88, 16.62)	(−1.91, 0.08)	(−8.16, 0.34)
**SDH**						
SDH in total population	8.64	36.92	25.59	109.32	−16.95	−72.40
	(7.51, 9.93)	(31.94, 41.89)	(23.49, 27.81)	(101.05, 117.59)	(NA)^1^	(NA)^1^
Recommended antihypertensive	8.64	36.92	22.24	95.00	−13.60	−58.08
medication in total population	(7.51, 9.93)	(31.94, 41.89)	(20.35, 24.25)	(86.82, 103.18)	(NA)^1^	(NA)^1^

*ACC/AHA, American College of Cardiology /American Heart Association; BP, Blood Pressure; CHL, Chinese Hypertension League Guideline; IDH, Isolated Diastolic Hypertension; ISH, Isolated Systolic Hypertension; NA, not applicable; SDH, Systolic and Diastolic Hypertension*.

*^1^ 95% CI cannot be estimated as SDH by CHL is subset of SDH by ACC/AHA*.

**Figure 2:**
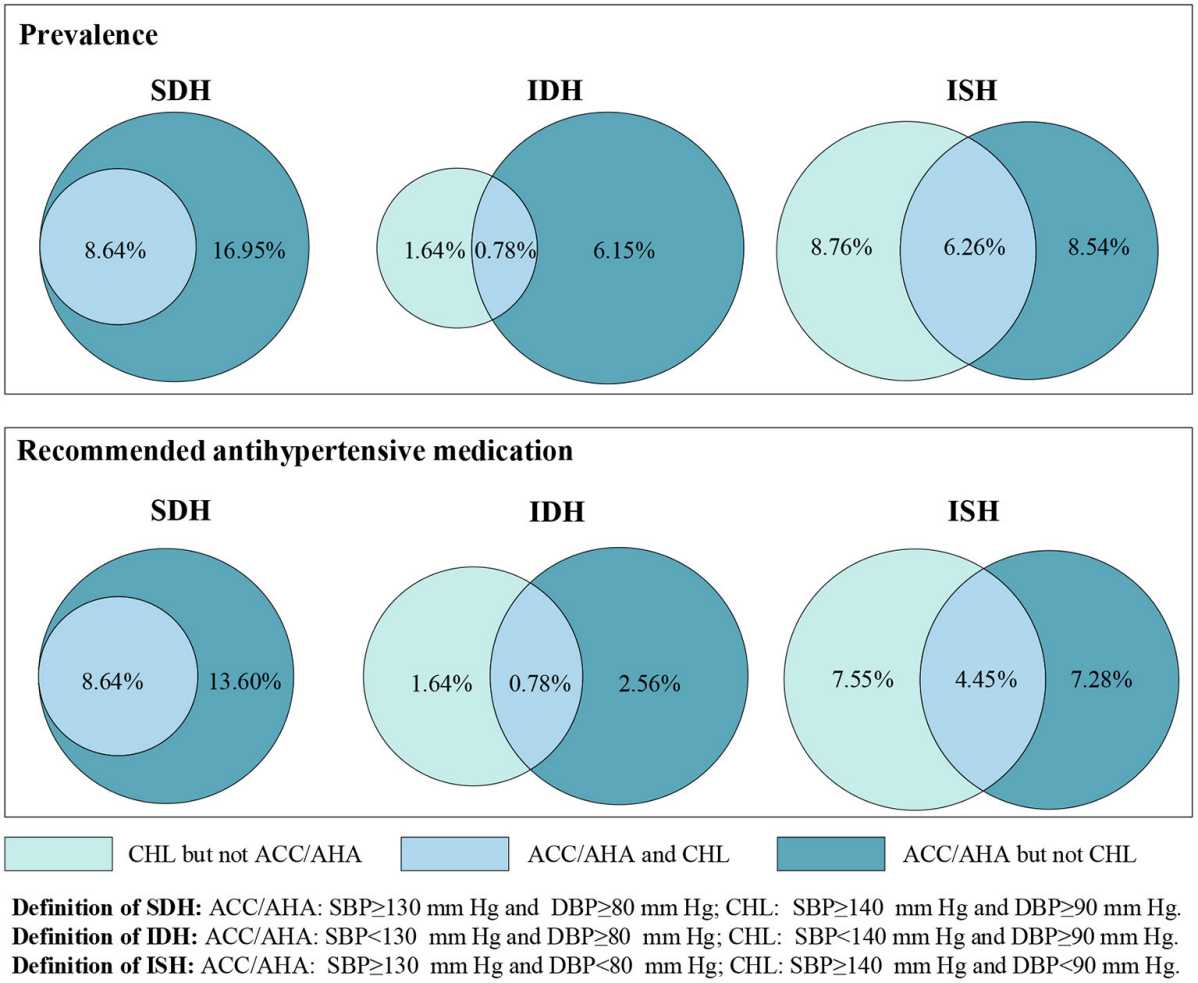
Percentage of Chinese adults who have ISH, IDH or SDH, recommended antihypertensive medication for ISH, IDH, or SDH defined by CHL only, ACC/AHA only, and both CHL and ACC/AHA.

A cross-tabulation of the prevalence for each of the categories of normotension, SDH, ISH and IDH between CHL and ACC/AHA definitions is shown in Appendix Table 3 and Figure 2. Overall, 8.64% (7.51, 9.93), 0.78% (0.33, 1.81) and 6.26% (5.54, 7.06) of the subjects met the definitions for SDH, ISH and IDH according to both guidelines. An additional 16.95% (15.24, 18.81), 6.15% (5.46, 6.91) and 8.54% (7.08, 10.27) were included based on ACC/AHA guideline only.

### Percentage and Number of Subjects Recommended for Antihypertensive Medication by Hypertension Subtypes

A total of 2.42% (1.54, 3.77) of Chinese adults were recommended for antihypertensive therapy for IDH by CHL, which is comparable to the 3.34% (2.64, 4.20) recommended by ACC/AHA. However, we observed a marked increase in the percentage and number of subjects recommended for drug therapy in SDH (13.60%, representing 58.08 million) (Table 1). Subgroup analysis between guidelines by sex and age group (≥65 years, <65 years) is shown in Appendix Table 2. Of note, a significant increase was observed for ISH (12.06%, representing 12.18 million) in subjects aged ≥65 years, which is in contrast to a decrease among those aged <65 years (−4.07%, representing 13.27 million) if ACC/AHA guideline was adopted.

Based on both definitions, 8.64, 0.78, and 4.45% of subjects were recommended to initiate antihypertensive medication for SDH, IDH and ISH, respectively. However, this corresponded to 13.60, 2.56, and 7.28% for ACC/AHA guideline only and 0, 1.64 and 7.55% for CHL guideline only (Figure 2).

### Associations Between Different Hypertension Subtypes and Mortality

After excluding 11,821 participants aged <18 years, 1,422 participants without BP measurements and 1,562 participants already undergoing antihypertensive treatment, 22,184 participants from the CHNS study were included in this analysis. Overall, the mean age was 40.86 years, and 45.12% were male. Regardless of the guidelines referenced, subjects with normotension were younger and had healthier cardiovascular status, such as lower body mass index. Moreover, compared to participants with ISH, those with IDH or SDH tended to be younger and male (Table 2).

**Table 2:** Descriptive baseline characteristics by different hypertension subtypes stratifying by 2018 CHL and 2017 ACC/AHA guidelines (from CHNS data).

	**2018 CHL guideline**				**2017 ACC/AHA guideline**				**Total**
	**Normotension**	**SDH**	**ISH**	**IDH**	**Normotension**	**SDH**	**ISH**	**IDH**	
Number of participants	18,838	1,293	884	1,169	12,434	3,768	816	5,166	22,184
Age, years	38.76 ± 14.58	55.47 ± 13.12	59.69 ± 13.67	44.24 ± 14.07	36.76 ± 13.94	52.79 ± 14.54	55.09 ± 16.63	39.78 ± 13.60	40.86 ± 15.47
**Age, %**									
<65	17,709(94.01)	967(74.79)	549(62.10)	1,076(92.04)	11,851(95.31)	2,960(78.56)	555(68.01)	4,935(95.53)	20,301(91.51)
≥65	1,129(5.99)	326(25.21)	335(37.90)	93(7.96)	583(4.69)	808(21.44)	261(31.99)	231(4.47)	1,883(8.49)
**Gender, %**									
Male	8,137(43.19)	730(56.46)	414(46.83)	728(62.28)	4,803(38.63)	2,068(54.88)	403(49.39)	2,735(52.94)	10,009(45.12)
Female	10,701(56.81)	563(43.54)	470(53.17)	441(37.72)	7,631(61.37)	1,700(45.12)	413(50.61)	2,431(47.06)	12,175(54.88)
**Educational level, %**									
Illiterate	3,762(20.49)	460(37.83)	349(42.05)	255(22.53)	2,533(20.84)	1,116(31.40)	281(36.12)	896(17.73)	4,826(22.40)
Primary school	3,345(18.22)	225(18.50)	148(17.83)	228(20.14)	21,77(17.91)	660(18.57)	154(19.79)	955(18.90)	39,46(18.32)
Middle/High school	9,562(52.07)	471(38.73)	292(35.18)	562(49.65)	6,355(52.28)	1,543(43.42)	294(37.79)	2,695(53.33)	1,0887(50.54)
Bachelor or above	1,694(9.23)	60(4.93)	41(4.94)	87(7.69)	1,091(8.97)	235(6.61)	49(6.30)	507(10.03)	1,882(8.74)
**Marital status, %**									
Never	2,522(13.56)	37(2.88)	26(2.96)	93(8.10)	1,882(15.34)	142(3.81)	51(6.34)	603(11.80)	2,678(12.22)
Married	16,080(86.44)	1,249(97.12)	851(97.04)	1,055(91.90)	1,0388(84.66)	3,586(96.19)	753(94.15)	4,508(88.20)	19,235(87.78)
**Registered residence, %**									
Urban	7,767(41.23)	559(43.23)	438(49.55)	451(38.58)	5,033(40.48)	1,666(44.21)	413(50.61)	2,103(40.71)	9,215(41.54)
Rural	11,071(58.77)	734(56.77)	446(50.45)	718(61.42)	7,401(59.52)	2,102(55.79)	403(49.39)	3,063(59.29)	12,969(58.46)
**Self-reported health excellent/very good, %**									
No	12,262(65.09)	855(66.13)	555(62.78)	727(62.19)	8,500(68.36)	2,343(62.18)	522(63.97)	3,034(58.73)	14,399(64.91)
Yes	3,576(34.91)	438(33.87)	329(37.22)	442(37.81)	3,934(31.64)	1,425(37.82)	294(36.03)	2,132(41.27)	7,785(35.09)
**Smoking status, %**									
Non-smoker	10,011(72.03)	735(62.24)	565(68.82)	561(59.43)	6,600(75.28)	2,188(64.75)	477(68.34)	2,607(65.18)	11,872(70.48)
Current smoker	3,668(26.39)	416(35.22)	219(26.67)	361(38.24)	2,064(23.54)	1,087(32.17)	200(28.65)	1,313(32.82)	4,664(27.69)
Ex-smoker	219(1.58)	30(2.54)	37(4.51)	22(2.33)	103(1.17)	104(3.08)	21(3.01)	80(2.00)	308(1.83)
**Drinking status, %**									
Non-drinker	9,385(67.17)	691(58.07)	563(67.34)	512(54.47)	6,219(70.57)	2,035(59.78)	487(68.98)	2,410(60.01)	1,1151(65.83)
Drinker	4,588(32.83)	499(41.93)	273(32.66)	428(45.53)	2,594(29.43)	1,369(40.22)	219(31.02)	1,606(39.99)	5,788(34.17)
**History of diseases, %**									
CVD	65(0.35)	13(1.01)	14(1.58)	7(0.60)	35(0.28)	36(0.96)	9(1.10)	19(0.37)	99(0.45)
Diabetes	185(0.98)	30(2.32)	30(3.39)	13(1.11)	89(0.72)	86(2.28)	35(4.29)	48(0.93)	258(1.16)
Cancer	67(0.36)	1(0.08)	5(0.57)	1(0.09)	39(0.31)	10(0.27)	10(1.23)	15(0.29)	74(0.33)
BMI, kg/m^2^	22.28 ± 3.32	24.45 ± 3.90	23.89 ± 3.80	23.94 ± 3.58	21.79 ± 2.96	24.16 ± 3.79	23.13 ± 3.58	23.15 ± 3.73	22.56 ± 3.45
SBP, mmHg	112.81 ± 11.77	155.00 ± 15.16	148.63 ± 10.28	127.37 ± 7.49	107.98 ± 10.11	143.12 ± 14.42	137.24 ± 9.37	118.46 ± 6.48	117.46 ± 16.81
DBP, mmHg	73.19 ± 8.04	98.14 ± 8.88	81.01 ± 6.32	92.04 ± 3.71	69.17 ± 6.47	90.00 ± 8.98	72.72 ± 5.90	82.53 ± 3.87	75.95 ± 10.57

*ACC/AHA, American College of Cardiology /American Heart Association; CHL, Chinese Hypertension League Blood Pressure Guideline; IDH, Isolated diastolic hypertension*.

*ISH, Isolated systolic hypertension; IDH, Isolated diastolic hypertension; SDH, Systolic-diastolic hypertension*.

**Figure 3:**
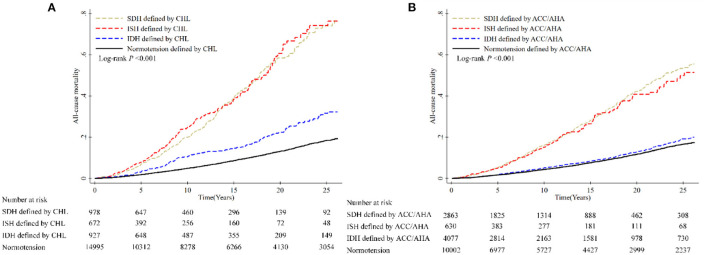
Cumulative incidence of all-cause mortality according to different definitions of hypertension. **(A)** Hypertension subtypes defined by CHL and all-cause mortality. **(B)** Hypertension subtypes defined by ACC/AHA and all-cause mortality. Data used for calculating hazard ratios of all-cause mortality were from CHNS.

**Figure 4:**
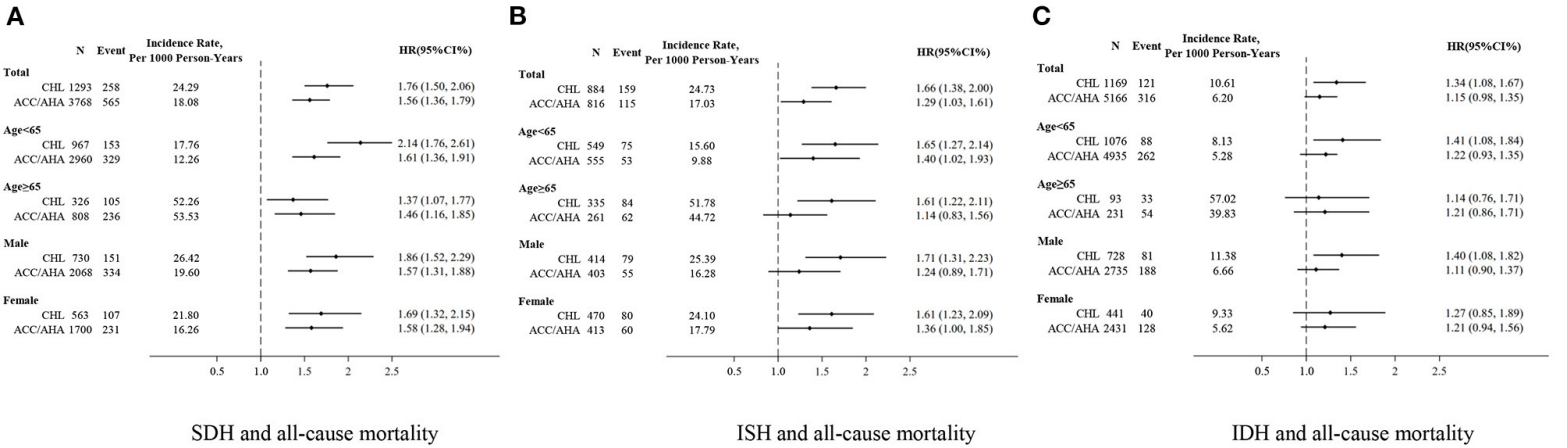
SDH, ISH and IDH are defined by CHL and ACC/AHA as associated with all-cause mortality. **(A)** SDH and all-cause mortality. **(B)** ISH and all-cause mortality. **(C)** IDH and all-cause mortality. SDH, ISH and IDH were defined as CHL compared to normotension defined as CHL (SBP <140 and DBP <90); SDH, ISH and IDH were defined as ACC/AHA compared to normotension defined as ACC/AHA (SBP <130 and DBP <80).

During a median follow-up of 6.14 (interquartile range, 2.02–17.98) years, 1,759 deaths occurred. The mortality rate (per 1,000 person-years) was 6.24 for normotension, 24.29 for SDH, 24.73 for ISH and 10.61 for IDH when the 2018 CHL definition was applied compared to rates of 5.64 for normal BP, 18.08 for SDH, 17.03 for ISH, and 6.20 for IDH according to the 2017 ACC/AHA guideline. Consistent with this, KM graphs by both definitions indicated a significant difference between hypertension subtypes (both *P* < 0.0001 by log-rank test), with a higher cumulative incidence of death for ISH and SDH, followed by IDH and normal BP (Figures 3A,B). However, the magnitude of the association was attenuated for hypertension subtypes defined using ACC/AHA guideline (HR 1.56 vs. 1.76 for SDH; 1.29 vs. 1.66 for ISH). Of note, we found no statistically significant association between IDH and death according to ACC/AHA guideline (1.15; 0.98, 1.35), which differs from estimates using CHL definition (1.34; 1.08, 1.67) (Figures 4A–C, Appendix Table 4).

In a series of sensitivity analyses, our results for premature death were similar to those for all-cause mortality; however, due to the lower rate of premature death, the level of uncertainty in these estimates was high (Appendix Figures 1A,B, Appendix Figures 2A,C, Appendix Table 4). The results were also similar to those of the overall population when stratified by age and sex (Appendix Figures 2A,C and Appendix Table 4). To test the reliability, we repeated analyses using a uniform reference group (optimal BP for both definitions: SBP <120 mm Hg and DBP <80 mm Hg) and did not identify material changes (Appendix Table 5). Finally, two additional sets of adjusted variables were included in the multivariable-adjusted Cox models. In general, the estimates were consistent with findings from fully adjusted models (Appendix Tables 4, 5).

## Discussion

Based on a national representative sample aged ≥45 years in CHARLS, we identified a substantial increase in hypertension prevalence by applying the 2017 ACC/AHA guideline, which was primarily manifested by a significant increase in SDH and IDH prevalence, particularly in the younger population. However, this marked rise was not observed for the eligibility of antihypertensive treatment in people with IDH. Additionally, except for estimates on SDH, the degree of agreement between both guidelines was minimal for the prevalence of IDH and ISH or the proportion of those recommended to initiate medication. Our study also identified a positive association between SDH and ISH but not IDH with all-cause mortality or premature death, although the magnitude of the association was attenuated if the 2017 ACC/AHA guideline was adopted. The positive association between different hypertension subtypes defined using the 2017 ACC/AHA guideline and CVD was confirmed by a meta-analysis but with a smaller effect size for IDH.

Our findings of the substantial increase in the percentage and number of subjects with hypertension are consistent with other reports from China and other countries (6–8, 10). However, in our analysis, we further demonstrated that the individual hypertension subtype distinctly contributed to the increase in terms of magnitude and direction. The most marked change stemmed from the increase in SDH followed by IDH, which contrasted with a relative decrease in ISH. Our findings are in accordance with data in the Korean population (10), although the rise in IDH prevalence is considered the major contributor to the increase in hypertension. We believe that this difference could be explained by the older population in our study (≥45 years), as previous evidence has indicated that IDH is commonly found in young participants (19, 20).

Several studies have reported the impact on the prevalence of recommended antihypertensive medication from the 2017 ACC/AHA guideline (5, 8, 21). In general, the increase is minimal [e.g., 1.9% for US adults (5) and 2.7% for Chinese adults (8)], although the corresponding absolute number differs markedly due to the population size of each country. However, few studies have further explored the impact of hypertension subtypes. In our study, unlike the significant increase in the prevalence of IDH, we did not observe this trend for the percentage of subjects who were recommended to initiate antihypertensive medications (2.42% for CHL vs. 3.34% for ACC/AHA). However, a significant increase of 12.06% in Chinese adults (12.18 million) was newly considered a candidate for initiating pharmacologic treatment in subjects aged ≥65 with ISH. This may be because most subjects being newly labeled as having IDH are younger adults who are unlikely to be candidates for drug therapy due to a lower predicted 10-year ASCVD risk, but this was the opposite in the elderly with ISH.

Our study observed a small proportion of subjects with ISH or IDH who met both guidelines, consistent with a study from John W. McEvoy et al. (22). This discordant population defined by CHL and ACC/AHA may affect these patients' prognostic risk. Several studies have demonstrated a high risk of mortality or CVD events with hypertension subtypes, the event for IDH, using 140/90 as the threshold for hypertension (23, 24). However, few studies have explored these associations with specific hypertension subtypes using the 2017 ACC/AHA definition, especially with respect to premature death. In our analysis of a cohort study with 22,184 participants, a significantly high total or premature mortality risk was found among subjects with SDH and ISH, regardless of the guidelines. However, this positive association was not observed among those with IDH defined using ACC/AHA guideline. Additionally, our study identified an increased CVD risk among subjects with SDH, ISH and IDH defined by the 2017 ACC/AHA through a meta-analysis. However, we found that most of the published reports addressed the relationship with IDH, but with an inconsistent conclusion from individual studies (22, 25–30). This was supported by a recent meta-analysis, from which a null association between IDH and CVD incidence according to the 2017 ACC/AHA guideline was reported among cohorts of middle-aged to older adults with rigorous BP measured (27).

Our study also has some limitations. First, our estimates on the percentage and number of subjects with different hypertension subtypes are based on CHARLS with a minimum age of 45 at baseline. This may limit our generalizability to subjects under 45 years old. Second, our study did not report the association of hypertension subtypes with cause-specific mortality, particularly cardiovascular death, although all-cause mortality as an endpoint could avoid misclassification issues compared to disease-specific mortality. Third, possible residual confounding could not be excluded due to the nature of our study design (i.e., observational study), even though a similar estimate was observed after performing several sensitivity analyses by including the covariates sequentially and using different reference groups.

In summary, our study undoubtedly revealed that hypertension prevalence or numbers will increase due to the lower BP threshold from the 2017 ACC/AHA guideline. We identified that SDH and IDH made a significant contribution to this increase. However, only a small increase was observed with respect to drug therapy initiation in subjects with IDH. Our further analysis indicated an attenuated mortality risk for ISH and SDH but a null association for IDH according to the 2017 ACC/AHA guideline.

## Data Availability Statement

The raw data supporting the conclusions of this article will be made available by the authors, without undue reservation.

## Ethics Statement

The studies involving human participants were reviewed and approved by the Xi'an Jiaotong University Health Science Centre (No: 2021-6). The patients/participants provided their written informed consent to participate in this study.

## Author Contributions

HM, CL, and TC was responsible for drafting the article and for overall content. KC and ZW assisted with the drafting and revision of the article. HM, CL, KC, ZW, FC, XL, YW, and TC provided inputs to the study concept and design, critically reviewed the results of analyses, and reviewed and contributed significantly to article revision. Statistical analyses were performed by CL and TC. TC provided full access to the study data as well as study oversight and article revision. All authors contributed to the article and approved the submitted version.

## Conflict of Interest

The authors declare that the research was conducted in the absence of any commercial or financial relationships that could be construed as a potential conflict of interest.

## Publisher's Note

All claims expressed in this article are solely those of the authors and do not necessarily represent those of their affiliated organizations, or those of the publisher, the editors and the reviewers. Any product that may be evaluated in this article, or claim that may be made by its manufacturer, is not guaranteed or endorsed by the publisher.
