# Supplementary material for: Hypertension Subtypes, Mortality Risk, and Differential Effects Between Two Hypertension Guidelines
Source: Front Med (Lausanne). 2022 Jul 5;9:814215. doi: 10.3389/fmed.2022.814215 (PMC9295617; doi:10.3389/fmed.2022.814215)
Supplement: Supplementary file 1 [file Data_Sheet_1.pdf]

Hypertension subtypes, mortality risk, and differential effect between two hypertension guidelines

Appendix methods

CHARLS is an ongoing nationally representative longitudinal survey of Chinese adults (age≥45 years). The baseline survey was conducted between June 2011 and March 2012. CHARLS covered 28 provinces, 150 counties/districts, and 450 village/urban communities across the country. The sampling strategy was through multistage probability sampling and stratified by region and within region by rural counties or urban districts and per capita statistics on gross domestic product, county-level units with the exception of Tibet were randomly chosen with a probability-proportional-to-size sampling technique. Furthermore, 3 PSUs were selected in each county and villages (rural)/neighborhoods (urban) were used as primary sampling units (PSUs). Detailed information including demographic characteristics, medical history, prescription drug use, clinical measurement and laboratory testing were collected by standard questionnaires[1].

CHNS is an ongoing longitudinal survey and used a multistage random-cluster sampling process to select samples from nine provinces across China for all age population and conducted from 1989 to 2015. There were nine provinces across four regions were included: East Coast region (Jiangsu and Shandong), Northeast region (Heilongjiang and Liaoning), West region (Guizhou and Gunagxi), Central region (Hennan, Hubei, and Hunan). Basically, nine selected provinces of CHNS vary according to geography, economic development and health indicators in China. CHNS used a weighted sampling scheme and were stratified by income (poor, middle and rich) to randomly select two cities and four counties in each province. Furthermore, 2 urban and 2 suburban neighborhoods within each city and one community in the capital city and three rural villages within each county were randomly selected. All subsequent surveys in CHNS have

obtained anthropometric, dietary, clinical, and all other individual data from each household member[2].

Appendix Table 1. Blood pressure threshold used to recommend antihypertensive medication according to the 2017 ACC/AHA Guideline and the 2018 CHL Guideline

Characteristic	2017 ACC/AHA	2018 CHL
**Systolic Blood Pressure**		
General population	≥140	≥140
Aged ≥65 years	≥130	≥150
High cardiovascular disease risk[1] or CKD	≥130	≥140
Diabetes	≥130	≥130
**Diastolic Blood Pressure**		
General population	≥90	≥90
Aged ≥65 years	≥80	≥90
High cardiovascular disease risk[1] or CKD	≥80	≥90
Diabetes	≥80	≥80

ACC/AHA, American College of Cardiology /American Heart Association; CHL, Chinese Hypertension League Blood Pressure Guideline

[1]High cardiovascular risk for ACC/AHA is defined as a history of cardiovascular disease (coronary heart disease, stroke or heart failure) or 10-year predicted cardiovascular disease risk of at least 10% using the pooled cohort risk equations.

Appendix Table 2. Comparison of Proportion, Numbers of SDH, ISH or IDH, recommended medication between CHL and ACC/AHA by age and sex[1]

Description	2018 CHL Guideline		2017 ACC/AHA Guideline		Difference between CHL and ACC/AHA	
	Proportion (95%CI) , %	Numbers (95%CI) , million	Proportion (95%CI) , %	Numbers (95%CI) , million	Proportion (95%CI) , %	Numbers (95%CI) , million
**Age<65**						
**SDH**						
SDH in total population	9.01 (7.61, 10.64)	29.41 (24.63, 34.18)	25.54 (22.87, 28.40)	83.37 (75.44, 91.30)	-16.53 NA	-53.96 NA
Recommended antihypertensive medication in total population	9.01 (7.61, 10.64)	29.41 (24.63, 34.18)	21.15 (18.83, 23.68)	69.05 (61.15, 76.95)	-12.14 NA	-39.58 NA
**ISH**						
ISH in total population	10.04 (8.12, 12.36)	32.78 (26.25, 39.31)	9.99 (8.43, 11.80)	32.61 (28.32, 36.91)	0.05 (-2.89, 2.99)	0.16 (-9.43, 9.76)
Recommended antihypertensive medication in total population	10.04 (8.12, 12.36)	32.78 (26.25, 39.31)	5.97 (5.27, 6.75)	19.49 (16.71, 22.26)	4.07 (1.80, 6.35)	13.27 (5.87, 20.70)
**IDH**						
IDH in total population	2.85 (1.81, 4.46)	9.29 (5.32, 13.27)	8.24 (7.20, 9.41)	26.90 (23.96, 29.83)	-5.39 (-6.91, -3.88)	-17.59 (-22.56,-12.67)
Recommended antihypertensive medication in total population	2.85 (1.81, 4.46)	9.29 (5.32, 13.27)	3.54 (2.81, 4.45)	11.56 (9.40, 13.71)	-0.69 (-1.97, 0.58)	-2.25 (-6.42, 1.89)
**Age≥65**						
**SDH**						
SDH in total population	7.45 (6.14, 9.02)	7.51 (6.15, 8.87)	25.76 (23.36, 28.31)	25.95 (23.59, 28.31)	-18.31 NA	-18.44 NA
Recommended antihypertensive medication in total population	7.45 (6.14, 9.02)	7.51 (6.15, 8.87)	25.76 (23.36, 28.31)	25.95 (23.59, 28.31)	-18.31 NA	-18.44 NA
**ISH**						
ISH in total population	31.16 (28.40, 34.06)	31.39 (28.79,33.99)	30.38 (27.98, 32.90)	30.61 (27.87, 33.35)	0.78 (-2.22, 3.77)	0.79 (-2.24,3.80)
Recommended antihypertensive medication in total population	18.32 (16.06, 20.83)	18.46 (16.15, 20.78)	30.38 (27.98, 32.90)	30.61 (27.87, 33.35)	-12.06 (-14.74, -9.38)	-12.18 (-14.89, -9.47)
**IDH**						
IDH in total population	1.03	1.04	2.67	2.69	-1.65	-1.67

	(0.42, 2.51)	(0.20, 1.87)	(1.79, 3.98)	(1.69, 3.70)	(-2.29, -1.00)	(-2.31, -1.01)
Recommended antihypertensive medication in total population	1.03<br>(0.42, 2.51)	1.04<br>(0.20, 1.87)	2.67<br>(1.79, 3.98)	2.69<br>(1.69, 3.70)	-1.65<br>(-2.29, -1.00)	-1.67<br>(-2.31, -1.01)
**Male**						
**SDH**						
SDH in total population	9.76<br>(8.54, 11.15)	20.11<br>(17.25, 22.97)	26.24<br>(24.15, 28.44)	54.04<br>(49.41, 58.66)	-16.48<br>NA	-33.93<br>NA
Recommended antihypertensive medication in total population	9.76<br>(8.53, 11.15)	20.11<br>(17.25, 22.97)	23.77<br>(21.60, 26.08)	48.95<br>(44.53, 53.37)	-14.01<br>NA	-28.86<br>NA
**ISH**						
ISH in total population	13.13<br>(11.56, 14.88)	27.05<br>(24.39, 29.70)	15.63<br>(12.72, 19.05)	32.18<br>(27.77, 36.60)	-2.50<br>(-6.45, 1.46)	-5.13<br>(-13.28, 3.01)
Recommended antihypertensive medication in total population	10.14<br>(8.76, 11.70)	20.88<br>(18.45, 23.31)	12.35<br>(10.82, 14.06)	25.43<br>(22.03, 28.84)	-2.21<br>(-4.30, -0.13)	-4.55<br>(-8.86, -0.27)
**IDH**						
IDH in total population	2.55<br>(1.26, 5.10)	5.26<br>(1.63,8.90)	8.23<br>(7.09, 9.53)	16.94<br>(14.83, 19.05)	-5.67<br>(-7.87, -3.47)	-11.68<br>(-16.21, -7.15)
Recommended antihypertensive medication in total population	2.55<br>(1.26, 5.10)	5.26<br>(1.63, 8.90)	4.34<br>(3.62, 5.20)	8.94<br>(7.43, 10.45)	-1.79<br>(-3.75, 0.18)	-3.69<br>(-7.73, 0.37)
**Female**						
**SDH**						
SDH in total population	7.60<br>(5.99, 9.60)	16.81<br>(12.72, 20.89)	24.99<br>(21.69, 28.61)	55.28<br>(48.40, 62.17)	-17.39<br>NA	-38.47<br>NA
Recommended antihypertensive medication in total population	7.60<br>(5.99, 9.60)	16.81<br>(12.72, 20.89)	20.82<br>(17.76, 24.24)	46.05<br>(39.10, 53.00)	-13.22<br>NA	-29.22<br>NA
**ISH**						
ISH in total population	16.78<br>(14.05, 19.92)	37.13<br>(30.87, 43.38)	14.03<br>(12.69, 15.49)	31.04<br>(28.27, 33.81)	2.75<br>(-0.49, 5.99)	6.09<br>(-1.08, 13.25)
Recommended antihypertensive medication in total population	13.73<br>(11.03, 16.95)	30.37<br>(24.05, 36.68)	11.15<br>(9.93,12.49)	24.66<br>(22.16, 27.16)	2.58<br>(-0.65, 5.81)	-5.70<br>(-1.44, 12.84)
**IDH**						
IDH in total population	2.29<br>(1.31, 3.98)	5.07<br>(3.23, 6.91)	5.72<br>(4.47, 7.30)	12.65<br>(10.40, 14.89)	-3.43<br>(-4.30, -2.55)	-7.58<br>(-9.51, -5.64)

Recommended antihypertensive medication in total population	2.29 (1.31, 3.98)	5.07 (3.23, 6.91)	2.40 (1.40, 4.09)	5.31 (3.47, 7.15)	-0.11 (-0.69, 0.47)	-0.24 (-1.52, 1.04)

ACC/AHA, American College of Cardiology /American Heart Association; BP, Blood Pressure; CHL, Chinese Hypertension League Blood Pressure Guideline; IDH, Isolated diastolic hypertension; ISH, Isolated systolic hypertension;

[1]Estimates of proportion and difference between CHL and ACC/AHA Blood Pressure Guideline from CHARLS data.

Appendix Table 3. Proportion of Chinese adults meeting the definitions for normotension, SDH, ISH, IDH according to 2017 ACC/AHA guideline and 2018 CHL guideline[1]

	2018 CHL Guideline			
	SBP<140 and DBP<90 mm Hg (Normotension)	SBP≥140 and DBP≥90 mm Hg (SDH)	SBP≥140 and DBP<90 mm Hg (ISH)	SBP<140 and DBP<90 mm Hg (IDH)
**2017 ACC/AHA Guideline**				
SBP<130 and DBP<80 mm Hg (Normotension)	52.68(50.06, 55.29)	NA	NA	NA
SBP≥130 and DBP≥80 mm Hg (SDH)	6.55(5.52, 7.75)	8.64(7.51, 9.93)	8.76(7.23, 10.58)	1.64(0.96, 2.79)
SBP≥130 and DBP<80 mm Hg (ISH)	8.54(7.08, 10.27)	NA	6.26(5.54, 7.06)	NA
SBP<130 and DBP≥80 mm Hg (IDH)	6.15(5.46, 6.91)	NA	NA	0.78(0.33, 1.81)

Abbreviation: ACC, American College of Cardiology; AHA, American Heart Association; CHL, Chinese Hypertension League; DBP, Diastolic Blood Pressure; IDH, Isolated Diastolic Hypertension; ISH, Isolated Systolic Hypertension; NA, not applicable; SBP, Systolic Blood Pressure; SDH, Systolic and Diastolic Hypertension.

[1]Numbers of Chinese Adults meeting each criterion of hypertension definition and 95%CI were reported.

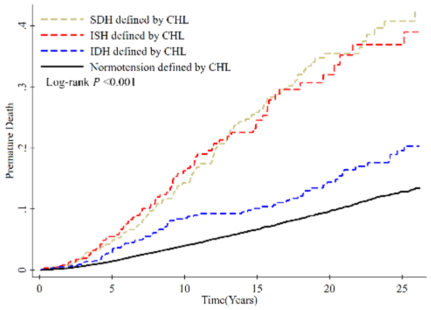

Number at risk	0	5	10	15	20	25
SDH defined by CHL	859	550	390	259	126	91
ISH defined by CHL	595	333	222	139	67	48
IDH defined by CHL	884	608	459	338	202	149
Normotension	14645	10019	8036	6103	4049	3046

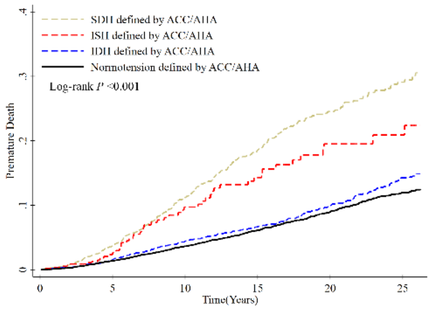

Number at risk	0	5	10	15	20	25
SDH defined by ACC/AHA	2612	1621	1173	805	434	306
ISH defined by ACC/AHA	565	334	240	161	105	68
IDH defined by ACC/AHA	4002	2749	2109	1548	960	729
Normotension	9804	6806	5585	4325	2945	2231

Appendix Figure 1A **Appendix Figure 1B**

Appendix Figure 1. Cumulative incidence of premature death according to different definition guideline of hypertension.

Data used for calculating Hazards Ratios of premature death were from CHNS.

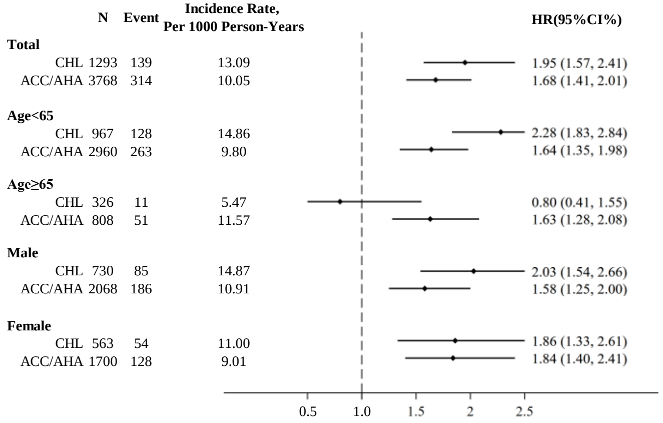

Appendix Figure 2A. SDH and premature death

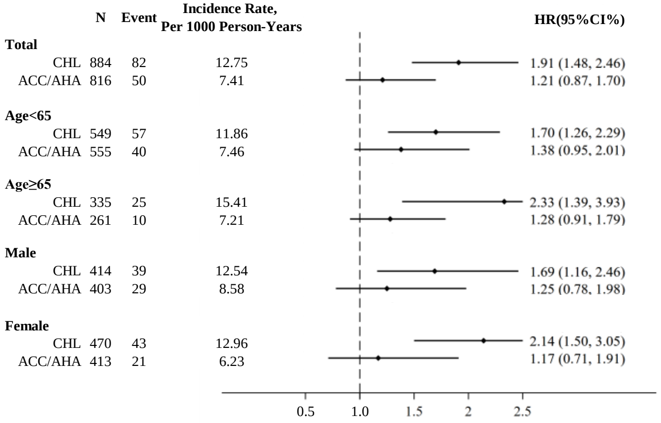

Appendix Figure 2B. ISH and premature death

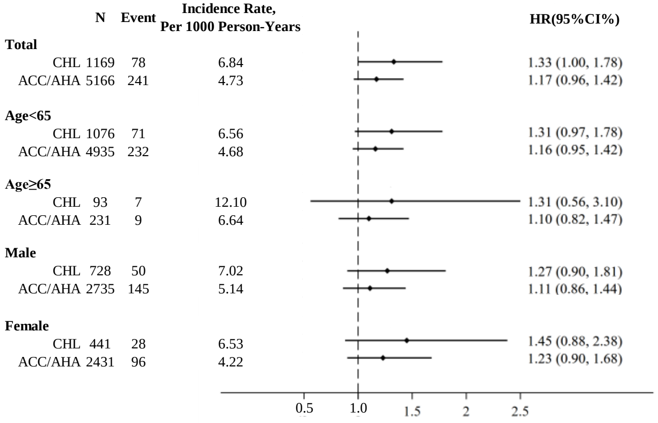

Appendix Figure 2C. IDH and premature death

Appendix Figure 2. SDH, ISH and IDH defined as CHL and ACC/AHA associated with Hazard Ratios of premature death.

SDH, ISH and IDH defined as CHL compared with Normotension defined as CHL (SBP<140 and DBP<90); SDH, ISH and IDH defined as ACC/AHA compared with Normotension defined as ACC/AHA (SBP<130 and DBP<80).

HRs adjusted for age, sex, educational level, marital status, whether rural residents, history of drinking or smoking, body mass index, previous history of diabetes mellitus, CVD or cancer.

Appendix Table 4. SDH, ISH and IDH associated with Hazard Ratios of all-cause mortality and premature death

	N	Event	Incidence Rate,	Model 1**		Model 2†		Model 3‡	
			Per 1000 Person-Years	HR	95%CI	HR	95%CI	HR	95%CI
**All-cause mortality**									
**CHL**									
Normotension	18838	1221	6.24	Reference		Reference		Reference	
SDH	1293	258	24.29	1.51	(1.31, 1.74)	1.56	(1.34, 1.82)	1.76	(1.50, 2.06)
ISH	884	159	24.73	1.30	(1.10, 1.54)	1.49	(1.24, 1.79)	1.66	(1.38, 2.00)
IDH	1169	121	10.61	1.19	(0.99, 1.44)	1.23	(0.99, 1.53)	1.34	(1.08, 1.67)
**ACC/AHA**									
Normotension	12434	763	5.64	Reference		Reference		Reference	
SDH	3768	565	18.08	1.30	(1.16, 1.45)	1.39	(1.22, 1.59)	1.56	(1.36, 1.79)
ISH	816	115	17.03	1.10	(0.90, 1.35)	1.26	(1.01, 1.57)	1.29	(1.03, 1.61)
IDH	5166	316	6.20	0.99	(0.87, 1.13)	1.07	(0.91, 1.25)	1.15	(0.98, 1.35)
**Premature death**									
**CHL**									
Normotension	18838	871	4.45	Reference		Reference		Reference	
SDH	1293	139	13.09	1.62	(1.35, 1.96)	1.72	(1.40, 2.12)	1.95	(1.57, 2.41)
ISH	884	82	12.75	1.45	(1.14, 1.83)	1.70	(1.32, 2.19)	1.91	(1.48, 2.46)
IDH	1169	78	6.84	1.20	(0.95, 1.52)	1.21	(0.91, 1.60)	1.33	(1.00, 1.78)
**ACC/AHA**									
Normotension	12434	565	4.18	Reference		Reference		Reference	
SDH	3768	314	10.05	1.37	(1.18, 1.59)	1.48	(1.24, 1.76)	1.68	(1.41, 2.01)
ISH	816	50	7.41	0.99	(0.74, 1.33)	1.19	(0.85, 1.65)	1.21	(0.87, 1.70)
IDH	5166	241	4.73	1.03	(0.88, 1.20)	1.07	(0.88, 1.29)	1.17	(0.96, 1.42)
**Age<65**									
**All cause mortality**									
**CHL**									
Normotension	17709	930	4.92	Reference		Reference		Reference	
SDH	967	153	17.76	1.83	(1.53, 2.18)	1.94	(1.60, 2.35)	2.14	(1.76, 2.61)
ISH	549	75	15.60	1.28	(1.01, 1.64)	1.51	(1.17, 1.95)	1.65	(1.27, 2.14)
IDH	1076	88	8.13	1.21	(0.97, 1.51)	1.29	(0.99, 1.67)	1.41	(1.08, 1.84)
**ACC/AHA**									

Normotension	11851	602	4.58	Reference		Reference		Reference	
SDH	2960	329	12.26	1.35	(1.17, 1.56)	1.47	(1.25, 1.73)	1.61	(1.36, 1.91)
ISH	555	53	9.88	1.17	(0.88, 1.55)	1.39	(1.01, 1.90)	1.40	(1.02, 1.93)
IDH	4935	262	5.28	1.01	(0.87, 1.16)	1.06	(0.88, 1.27)	1.12	(0.93, 1.35)
**Premature death**									
**CHL**									
Normotension	17709	802	4.24	Reference		Reference		Reference	
SDH	967	128	14.86	1.94	(1.60, 2.35)	2.04	(1.64, 2.52)	2.28	(1.83, 2.84)
ISH	549	57	11.86	1.31	(0.99, 1.73)	1.53	(1.14, 2.05)	1.70	(1.26, 2.29)
IDH	1076	71	6.56	1.17	(0.92, 1.49)	1.18	(0.87, 1.59)	1.31	(0.97, 1.78)
**ACC/AHA**									
Normotension	11851	523	3.98	Reference		Reference		Reference	
SDH	2960	263	9.80	1.37	(1.17, 1.60)	1.46	(1.21, 1.75)	1.64	(1.35, 1.98)
ISH	555	40	7.46	1.13	(0.82, 1.56)	1.37	(0.95, 1.97)	1.38	(0.95, 2.01)
IDH	4935	232	4.68	1.03	(0.88, 1.20)	1.08	(0.88, 1.32)	1.16	(0.95, 1.42)
**Age≥65**									
**All cause mortality**									
**CHL**									
Normotension	1129	291	43.69	Reference		Reference		Reference	
SDH	326	105	52.26	1.21	(0.97, 1.51)	1.21	(0.95, 1.55)	1.37	(1.07, 1.77)
ISH	335	84	51.78	1.31	(1.03, 1.67)	1.43	(1.09, 1.87)	1.61	(1.22, 2.11)
IDH	93	33	57.02	1.12	(0.78, 1.62）	1.10	(0.74, 1.64)	1.14	(0.76, 1.71)
**ACC/AHA**									
Normotension	583	161	43.30	Reference		Reference		Reference	
SDH	808	236	53.53	1.23	(1.00, 1.51)	1.30	(1.04, 1.63)	1.46	(1.16, 1.85)
ISH	261	62	44.72	0.99	(0.74, 1.33)	1.11	(0.81, 1.52)	1.14	(0.83, 1.56)
IDH	231	54	39.83	0.94	(0.68, 1.28)	1.09	(0.78, 1.53)	1.21	(0.86, 1.71)
**Premature death**									
**CHL**									
Normotension	1129	69	10.36	Reference		Reference		Reference	
SDH	326	11	5.47	0.54	(0.29, 1.03)	0.66	(0.34, 1.27)	0.80	(0.41, 1.55)
ISH	335	25	15.41	1.45	(0.92, 2.29)	1.83	(1.10, 3.04)	2.33	(1.39, 3.93)
IDH	93	7	12.10	1.22	(0.56, 2.66)	1.29	(0.55, 3.02)	1.31	(0.56, 3.10）

**ACC/AHA**								
Normotension	583	42	11.29	Reference		Reference		Reference	
SDH	808	51	11.57	1.18	(0.75, 1.86)	1.45	(0.91, 2.33)	1.63	(1.28, 2.08)
ISH	261	10	7.21	0.80	(0.38, 1.67)	0.94	(0.45, 2.00)	1.28	(0.91, 1.79)
IDH	231	9	6.64	0.47	(0.20, 1.12)	0.53	(0.22, 1.27)	1.10	(0.82, 1.47)
**Male**									
**All cause mortality**									
**CHL**									
Normotension	8137	692	7.58	Reference		Reference		Reference	
SDH	730	151	26.42	1.53	(1.27, 1.83)	1.67	(1.36, 2.04)	1.86	(1.52, 2.29)
ISH	414	79	25.39	1.26	(0.99, 1.60)	1.55	(1.19, 2.01)	1.71	(1.31, 2.23)
IDH	728	81	11.38	1.17	(0.93, 1.47)	1.32	(1.01, 1.71)	1.40	(1.08, 1.82)
**ACC/AHA**									
Normotension	4803	426	7.27	Reference		Reference		Reference	
SDH	2068	334	19.60	1.27	(1.09, 1.48)	1.43	(1.20, 1.70)	1.57	(1.31, 1.88)
ISH	403	55	16.28	1.01	(0.76, 1.35)	1.17	(0.85, 1.62)	1.24	(0.89, 1.71)
IDH	2735	188	6.66	0.91	(0.77, 1.08)	1.03	(0.84, 1.27)	1.11	(0.90, 1.37)
**Premature death**									
**CHL**									
Normotension	8137	501	5.49	Reference		Reference		Reference	
SDH	730	85	14.87	1.63	(1.28, 2.08)	1.81	(1.39, 2.37)	2.03	(1.54, 2.66)
ISH	414	39	12.54	1.28	(0.91, 1.79)	1.53	(1.06, 2.22)	1.69	(1.16, 2.46)
IDH	728	50	7.02	1.10	(0.82, 1.47)	1.19	(0.84, 1.68)	1.27	(0.90, 1.81)
**ACC/AHA**									
Normotension	4803	315	5.38	Reference		Reference		Reference	
SDH	2068	186	10.91	1.31	(1.08, 1.59)	1.43	(1,14, 1.80)	1.58	(1,25, 2.00)
ISH	403	29	8.58	1.05	(0.71, 1.54)	1.22	(0.77, 1.91)	1.25	(0.78, 1.98)
IDH	2735	145	5.14	0.94	(0.77, 1.14)	1.03	(0.80, 1.33)	1.11	(0.86, 1.44)
**Female**									
**All cause mortality**									
**CHL**									
Normotension	10701	529	5.06	Reference		Reference		Reference	
SDH	563	107	21.80	1.49	(1.21, 1.85)	1.47	(1.16, 1.86)	1.69	(1.32, 2.15)

ISH	470	80	24.10	1.33	(1.04, 1.69)	1.43	(1.10, 1.85)	1.61	(1.23, 2.09)
IDH	441	40	9.33	1.25	(0.90, 1.72)	1.10	(0.74, 1.64)	1.27	(0.85, 1.89)
**ACC/AHA**									
Normotension	7631	337	4.40	Reference		Reference		Reference	
SDH	1700	231	16.26	1.33	(1.11, 1.59)	1.37	(1.12, 1.67)	1.58	(1.28, 1.94)
ISH	413	60	17.79	1.19	(0.90, 1.58)	1.34	(0.99, 1.82)	1.36	(1.00, 1.85)
IDH	2431	128	5.62	1.11	(0.91, 1.37)	1.12	(0.87, 1.43)	1.21	(0.94, 1.56)
**Premature death**									
**CHL**									
Normotension	10701	370	3.54	Reference		Reference		Reference	
SDH	563	54	11.00	1.61	(1.19, 2.16)	1.62	(1.17, 2.26)	1.86	(1.33, 2.61)
ISH	470	43	12.96	1.64	(1.18, 2.29)	1.86	(1.31, 2.65)	2.14	(1.50, 3.05)
IDH	441	28	6.53	1.42	(0.97, 2.09)	1.23	(0.75, 2.01)	1.45	(0.88, 2.38)
**ACC/AHA**									
Normotension	7631	250	3.26	Reference		Reference		Reference	
SDH	1700	128	9.01	1.45	(1.15, 1.82)	1.54	(1.18, 2.00)	1.84	(1.40, 2.41)
ISH	413	21	6.23	0.92	(0.58, 1.45)	1.15	(0.71, 1.86)	1.17	(0.71, 1.91)
IDH	2431	96	4.22	1.17	(0.92, 1.48)	1.10	(0.81, 1.49)	1.23	(0.90, 1.68)

ACC/AHA, American College of Cardiology /American Heart Association; BP, Blood Pressure; CHL, Chinese Hypertension League Blood Pressure Guideline; IDH, Isolated diastolic hypertension; ISH, Isolated systolic hypertension.

[1]ISH was defined as SBP≥140 and DBP<90; IDH was defined as SBP<140 and DBP≥90.

[2]ISH was defined as SBP≥130 and DBP<80; IDH was defined as SBP<130 and DBP≥80.

[3]Data used for calculating Hazards Ratios of all-cause mortality and premature death were from CHARLS.

**Model 1: adjusted for age and sex.

†Model 2: adjusted for age, sex, educational level, marital status, whether rural residents, history of drinking or smoking.

‡Model 3: adjusted for age, sex, educational level, marital status, whether rural residents, history of drinking or smoking, body mass index, previous history of diabetes mellitus, CVD or cancer.

Appendix Table 5. SDH, ISH and IDH associated with Hazard Ratios of all-cause mortality and premature death with optimal BP as reference group*

	N	Event	Incidence Rate,	Model 1**		Model 2†		Model 3‡	
			Per 100 Person-Years	HR	95%CI	HR	95%CI	HR	95%CI
**All-cause mortality**									
**CHL**									
Optimal	10342	609	5.25	Reference		Reference		Reference	
SDH	1293	258	24.29	1.53	(1.31, 1.78)	1.60	(1.35, 1.90)	1.87	(1.57, 2.23)
ISH	884	159	24.73	1.31	(1.10, 1.58)	1.53	(1.25, 1.87)	1.76	(1.44, 2.16)
IDH	1169	121	10.61	1.20	(0.99, 1.47)	1.26	(1.00, 1.58)	1.42	(1.13, 1.80)
**ACC/AHA**									
Optimal	10342	609	5.25	Reference		Reference		Reference	
SDH	3768	565	18.08	1.31	(1.16, 1.48)	1.39	(1.21, 1.60)	1.58	(1.36, 1.82)
ISH	816	115	17.03	1.12	(0.91, 1.37)	1.26	(1.01, 1.58)	1.30	(1.04, 1.64)
IDH	5166	316	6.20	1.01	(0.88, 1.15)	1.07	(0.90, 1.26)	1.16	(0.98, 1.37)
**Premature death**									
**CHL**									
Optimal	10342	466	4.02	Reference		Reference		Reference	
SDH	1293	139	13.09	1.64	(1.34, 2.00)	1.72	(1.37, 2.16)	2.04	(1.61, 2.57)
ISH	884	82	12.75	1.46	(1.14, 1.87)	1.70	(1.30, 2.23)	2,00	(1.52, 2.63)
IDH	1169	78	6.84	1.21	(0.96, 1.55)	1.21	(0.90, 1.63)	1.39	(1.03, 1.89)
**ACC/AHA**									
Optimal	10342	466	4.02	Reference		Reference		Reference	
SDH	3768	314	10.05	1.38	(1.18, 1.61)	1.45	(1.21, 1.74)	1.67	(1.39, 2.02)
ISH	816	50	7.41	1.00	(0.74, 1.35)	1.17	(0.83, 1.63)	1.20	(0.85, 1.70)
IDH	5166	241	4.73	1.03	(0.88, 1.21)	1.05	(0.86, 1.28)	1.16	(0.95, 1.42)
**Age<65**									
**All cause mortality**									
**CHL**									
Optimal	9961	493	4.35	Reference		Reference		Reference	
SDH	967	153	17.76	1.85	(1.53, 2.23)	1.96	(1.59, 2.42)	2.22	(1.79, 2.76)
ISH	549	75	15.60	1.30	(1.01, 1.67)	1.53	(1.16, 2.00)	1.71	(1.30, 2.25)
IDH	1076	88	8.13	1.22	(0.97, 1.54)	1.30	(0.99, 1.71)	1.47	(1.11, 1.94)

**ACC/AHA**									
Optimal	9961	493	4.35	Reference		Reference		Reference	
SDH	2960	329	12.26	1.37	(1.19, 1.59)	1.47	(1.24, 1.74)	1.62	(1.36, 1.93)
ISH	555	53	9.88	1.19	(0.89, 1.58)	1.39	(1.01, 1.91)	1.41	(1.02, 1.95)
IDH	4935	262	5.28	1.02	(0.88, 1.18)	1.06	(0.88, 1.28)	1.13	(0.93, 1.36)
**Premature death**									
**CHL**									
Optimal	9961	434	3.83	Reference		Reference		Reference	
SDH	967	128	14.86	1.95	(1.59, 2.40)	2.03	(1.60, 2.56)	2.35	(1.85, 3.00)
ISH	549	57	11.86	1.32	(0.99, 1.75)	1.52	(1.12, 2.07)	1.75	(1.28, 2.40)
IDH	1076	71	6.56	1.17	(0.91, 1.51)	1.17	(0.86, 1.61)	1.35	(0.98, 1.86)
**ACC/AHA**									
Optimal	9961	434	3.83	Reference		Reference		Reference	
SDH	2960	263	9.80	1.38	(1.17, 1.62)	1.43	(1.18, 1.74)	1.62	(1.33, 1.98)
ISH	555	40	7.46	1.14	(0.82, 1.58)	1.35	(0.93, 1.95)	1.37	(0.94, 2.01)
IDH	4935	232	4.68	1.03	(0.88, 1.21)	1.06	(0.86, 1.31)	1.15	(0.93, 1.42)
**Age≥65**									
**All cause mortality**									
**CHL**									
Optimal	381	116	44.06	Reference		Reference		Reference	
SDH	326	105	52.26	1.21	(0.92, 1.57)	1.28	(0.96, 1.72)	1.54	(1.14, 2.09)
ISH	335	84	51.78	1.30	(0.98, 1.73)	1.51	(1.11, 2.08)	1.80	(1.31, 2.48)
IDH	93	33	57.02	1.12	(0.75, 1.66)	1.16	(0.76, 1.79)	1.28	(0.83, 1.97)
**ACC/AHA**									
Optimal	381	116	44.06	Reference		Reference		Reference	
SDH	808	236	53.53	1.23	(0.98, 1.55)	1.32	(1.03, 1.70)	1.54	(1.19, 2.01)
ISH	261	62	44.72	0.99	(0.73, 1.35)	1.13	(0.81, 1.58)	1.20	(0.86, 1.68)
IDH	231	54	39.83	0.94	(0.68, 1.30)	1.11	(0.78, 1.59)	1.28	(0.89, 1.84)
**Premature death**									
**CHL**									
Optimal	381	32	12.16	Reference		Reference		Reference	
SDH	326	11	5.47	0.48	(0.24, 0.96)	0.59	(0.29, 1.19)	0.76	(0.37, 1.58)
ISH	335	25	15.41	1.29	(0.76, 2.17)	1.62	(0.90, 2.90)	2.22	(1.21, 4.08)

	IDH	93	7	12.10	1.08	(0.47, 2.46)	1.14	(0.46, 2.81)	1.25	(0.50, 3.11)
**ACC/AHA**										
	Optimal	381	32	12.16	Reference		Reference		Reference	
	SDH	808	51	11.57	1.02	(0.65, 1.59)	1.13	(0.69, 1.85)	1.44	(0.86, 2.42)
	ISH	261	10	7.21	0.66	(0.33, 1.35)	0.77	(0.36, 1.65)	0.94	(0.43, 2.04)
	IDH	231	9	6.64	0.53	(0.25, 1.11)	0.45	(0.18, 1.10)	0.52	(0.21, 1.29)
**Male**										
**All cause mortality**										
**CHL**										
	Optimal	3766	333	6.88	Reference		Reference		Reference	
	SDH	730	151	26.42	1.52	(1.24, 1.86)	1.71	(1.36, 2.15)	1.98	(1.56, 2.50)
	ISH	414	79	25.39	1.26	(0.97, 1.62)	1.59	(1.19, 2.11)	1.81	(1.36, 2.42)
	IDH	728	81	11.38	1.16	(0.91, 1.48)	1.35	(1.02, 1.79)	1.48	(1.11, 1.97)
**ACC/AHA**										
	Optimal	3766	333	6.88	Reference		Reference		Reference	
	SDH	2068	334	19.60	1.31	(1.11, 1.53)	1.46	(1.21, 1.77)	1.61	(1.32, 1.95)
	ISH	403	55	16.28	1.04	(0.78, 1.39)	1.20	(0.86, 1.67)	1.27	(0.91, 1.77)
	IDH	2735	188	6.66	0.93	(0.78, 1.12)	1.06	(0.85, 1.31)	1.14	(0.91, 1.42)
**Premature death**										
**CHL**										
	Optimal	3766	255	5.27	Reference		Reference		Reference	
	SDH	730	85	14.87	1.62	(1.25, 2.10)	1.79	(1.33, 2.41)	2.07	(1.52, 2.81)
	ISH	414	39	12.54	1.26	(0.89, 1.79)	1.52	(1.03, 2.25)	1.73	(1.16, 2.57)
	IDH	728	50	7.02	1.09	(0.80, 1.48)	1.17	(0.81, 1.70)	1.30	(1.02, 1.04)
**ACC/AHA**										
	Optimal	3766	255	5.27	Reference		Reference		Reference	
	SDH	2068	186	10.91	1.32	(1.08, 1.61)	1.41	(1.10, 1.79)	1.55	(1.21, 2.00)
	ISH	403	29	8.58	1.05	(0.71, 1.56)	1.20	(0.75, 1.90)	1.23	(0.77, 1.97)
	IDH	2735	145	5.14	0.94	(0.77, 1.16)	1.02	(0.78, 1.32)	1.10	(0.84, 1.44)
**Female**										
**All cause mortality**										
**CHL**										
	Optimal	6576	276	4.08	Reference		Reference		Reference	

	SDH	563	107	21.80	1.53	(1.21, 1.93)	1.50	(1.16, 1.95)	1.79	(1.37, 2.35)
	ISH	470	80	24.10	1.36	(1.05, 1.77)	1.46	(1.10, 1.94)	1.71	(1.28, 2.28)
	IDH	441	40	9.33	1.27	(0.91, 1.78)	1.13	(0.75, 1.70)	1.34	(0.89, 2.04)
**ACC/AHA**										
	Optimal	6576	276	4.08	Reference		Reference		Reference	
	SDH	1700	231	16.26	1.32	(1.10, 1.59)	1.33	(1.08, 1.64)	1.56	(1.25, 1.94)
	ISH	413	60	17.79	1.18	(0.89, 1.58)	1.31	(0.96, 1.78)	1.34	(0.98, 1.84)
	IDH	2431	128	5.62	1.11	(0.90, 1.37)	1.09	(0.84, 1.41)	1.20	(0.92, 1.55)
**Premature death**										
**CHL**										
	Optimal	6576	211	3.12	Reference		Reference		Reference	
	SDH	563	54	11.00	1.65	(1.21, 2.27)	1.63	(1.14, 2.33)	1.99	(1.37, 2.87)
	ISH	470	43	12.96	1.69	(1.19, 2.40)	1.88	(1.29, 2.74)	2.28	(1.55, 3.36)
	IDH	441	28	6.53	1.46	(0.98, 2.17)	1.24	(0.74, 2.06)	1.54	(0.92, 2.58)
**ACC/AHA**										
	Optimal	6576	211	3.12	Reference		Reference		Reference	
	SDH	1700	128	9.01	1.45	(1.14, 1.84)	1.50	(1.14, 1.98)	1.83	(1.37, 2.43)
	ISH	413	21	6.23	0.92	(0.58, 1.46)	1.12	(0.69, 1.83)	1.16	(0.70, 1.91)
	IDH	2431	96	4.22	1.17	(0.92, 1.49)	1.08	(0.79, 1.47)	1.23	(0.89, 1.69)

ACC/AHA, American College of Cardiology /American Heart Association; BP, Blood Pressure; CHL, Chinese Hypertension League Blood Pressure Guideline; IDH, Isolated diastolic hypertension; ISH, Isolated systolic hypertension.

*Data used for calculating Hazards Ratios of all-cause mortality and premature death were from CHARLS.

**Model 1: adjusted for age and sex.

†Model 2: adjusted for age, sex, educational level, marital status, whether rural residents, history of drinking or smoking.

‡Model 3: adjusted for age, sex, educational level, marital status, whether rural residents, history of drinking or smoking, body mass index, previous history of diabetes mellitus, CVD or cancer.

1. Zhao Y, Hu Y, Smith JP, Strauss J, Yang G. Cohort profile: the China Health and Retirement Longitudinal Study (CHARLS). *Int J Epidemiol.* 2014;43(1):61-68. doi: 10.1093/ije/dys203
2. Popkin BM, Du S, Zhai F, Zhang B. Cohort Profile: The China Health and Nutrition Survey--monitoring and understanding socio-economic and health change in China, 1989-2011. *Int J Epidemiol.* 2010;39(6):1435-1440. doi: 10.1093/ije/dyp322
